# Immunization with matrix-, nucleoprotein and neuraminidase protects against H3N2 influenza challenge in pH1N1 pre-exposed pigs

**DOI:** 10.1038/s41541-023-00620-2

**Published:** 2023-02-15

**Authors:** Eleni Vatzia, Katherine Feest, Adam McNee, Tanuja Manjegowda, B. Veronica Carr, Basudev Paudyal, Tiphany Chrun, Emmanuel A. Maze, Amy Mccarron, Susan Morris, Helen E. Everett, Ronan MacLoughlin, Francisco J. Salguero, Teresa Lambe, Sarah C. Gilbert, Elma Tchilian

**Affiliations:** 1 The Pirbright Institute, Pirbright, United Kingdom; 2 Nuffield Department of Medicine, University of Oxford, Oxford, United Kingdom; 3 Animal and Plant Health Agency-Weybridge, New Haw, Addlestone, United Kingdom; 4 Aerogen Ltd, Galway, Ireland; 5 United Kingdom Health Security Agency, UKHSA-Porton Down, Salisbury, United Kingdom; 6 Oxford Vaccine Group, Department of Paediatrics, Medical Sciences Division, University of Oxford and Chinese Academy of Medical Science (CAMS) Oxford Institute (COI), University of Oxford, Oxford, United Kingdom

**Keywords:** Vaccines, Influenza virus, Vaccines

## Abstract

There is an urgent need for influenza vaccines providing broader protection that may decrease the need for annual immunization of the human population. We investigated the efficacy of heterologous prime boost immunization with chimpanzee adenovirus (ChAdOx2) and modified vaccinia Ankara (MVA) vectored vaccines, expressing conserved influenza virus nucleoprotein (NP), matrix protein 1 (M1) and neuraminidase (NA) in H1N1pdm09 pre-exposed pigs. We compared the efficacy of intra-nasal, aerosol and intra-muscular vaccine delivery against H3N2 influenza challenge. Aerosol prime boost immunization induced strong local lung T cell and antibody responses and abrogated viral shedding and lung pathology following H3N2 challenge. In contrast, intramuscular immunization induced powerful systemic responses and weak local lung responses but also abolished lung pathology and reduced viral shedding. These results provide valuable insights into the development of a broadly protective influenza vaccine in a highly relevant large animal model and will inform future vaccine and clinical trial design.

## Introduction

Respiratory viruses are a major threat to global human health with influenza infection responsible for seasonal epidemics and occasional pandemics. Current seasonal influenza vaccines primarily induce antibody responses to the major envelope glycoprotein haemagglutinin (HA), are strain specific and do not protect well against antigenically distinct viruses from the same HA subtype nor against infection with heterologous influenza viruses from different HA lineages. A broadly protective influenza A vaccine (BPIV) would be a significant advance in preventing seasonal infection and reducing mortality from pandemic influenza.

Although most influenza vaccines are administered parenterally to adults, the intranasally administered live attenuated influenza vaccine (LAIV) shows high efficacy in children suggesting that both systemic and local respiratory immunity play roles in protection^1,2^. Inhaled aerosol delivery methods have been explored to administer measles, tuberculosis, papilloma virus vaccines, and recently, the safety and immunogenicity of intranasal and aerosolized vaccines has been investigated in pre-clinical and clinical studies for SARS-CoV-2^3–11^. Comparison of aerosol, intranasal and parenteral delivery of an adenovirus-vectored vaccine in rhesus macaques indicated that each route generated distinct cellular and humoral responses and suggested that small droplet aerosol delivery offered immunological advantages over other respiratory routes^12^.

In addition, when considering immunization against influenza, it is important to take into account that pre-existing immune memory can significantly impact subsequent influenza infection and vaccine efficacy^13,14^. Cross-reactive immunity acquired by prior seasonal influenza infections is principally due to T cell responses to conserved internal antigens NP and M1 and antibodies to conserved epitopes of the haemagglutinin (HA) and neuraminidase (NA) are likely to play a role^15–19^. Approaches toward development of BPIV have therefore focussed on either eliciting antibodies against the conserved regions of HA and NA or stimulating T cells against the internal NP and M1 proteins of the virus^20–29^. We have previously evaluated the efficacy of a single cycle replication BPIV candidate, S-FLU, in pigs^30,31^. These studies have shown that vaccine efficacy in pigs differed from that in small animals (ferrets and mice), suggesting that vaccines should be tested in more than one animal model^30^. Pigs are immunologically, physiologically and anatomically more like humans than small laboratory animals and have a comparable distribution of sialic acid receptors in the respiratory tract. Pigs exhibit comparable clinical manifestations and pathogenesis when infected with most human seasonal influenza A viruses, making them an excellent model to study immunity to influenza^32,33^.

Because pigs are susceptible to the influenza A viruses that infect humans, we have developed an H1N1pdm09 (pH1N1) 1A.3.3.2 virus pre-exposure model to test the immunogenicity of a ChAdOx2 viral vectored vaccine, expressing influenza nucleoprotein, matrix protein 1 from pH1N1 and neuraminidase NA2 from A/swine/Ohio/A01354299/2017, H3N2 (ChAdOx2-NPM1-NA2)^34^. We have shown that previous influenza virus pH1N1 infection in pigs does not prevent induction of immune responses following immunization with ChAdOx2-NPM1-NA2^34^. We have also evaluated the importance of route of administration by comparing intra-nasal, aerosol and intra-muscular immunization. In these studies, aerosol delivery boosted the local lung T cell and antibody responses, while intra-muscular immunization boosted peripheral blood immunity^34^. However, protective efficacy was not evaluated and therefore in the present study we assessed the immunogenicity and efficacy of prime boost with ChAdOx2-NPM1-NA2 and MVA-NPM1-NA2 administered by the intra-muscular, intra-nasal or aerosol routes in pH1N1 pre-exposed pigs against H3N2 challenge.

## Results

### Experimental design and antibody responses following prime boost immunization with ChAdOx2-NPM1-NA2 and MVA-NPM1-NA2 in pH1N1 pre-exposed pigs

**Fig. 1:**
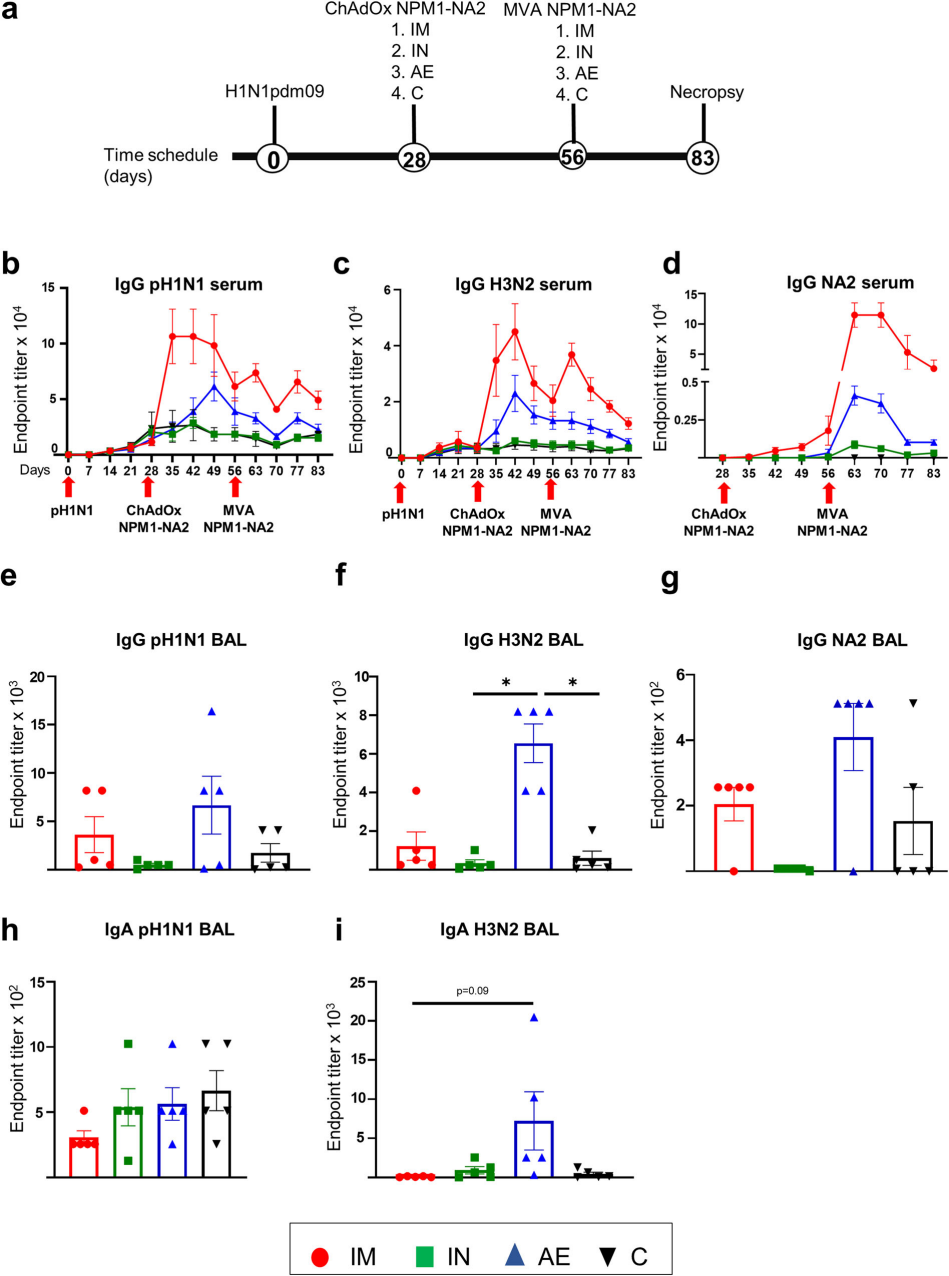
Experimental design and antibody responses. Twenty pigs were infected with pH1N1 and four weeks later were immunized with ChAdOx-NPM1-NA2 intramuscularly (IM), intranasally (IN) or by aerosol (AE). Four weeks later they were boosted with MVA-NPM1-NA2 and after further four weeks were culled. Weekly blood samples were collected. Control (C) animals were infected but not immunized (**a**). pH1N1 (**b**), H3N2 (**c**) and NA2 (**d**) specific IgG responses in serum were determined by ELISA at the indicated time points. pH1N1 (**e**), H3N2 (**f**) and NA2 (**g**) specific IgG and pH1N1 (**h**) and H3N2 (**i**) IgA responses in BAL, were determined by ELISA four weeks after the second immunization. The mean and standard error (SEM) is presented in each time point (**b**–**d**). The arrows below D0, D28 and D56 indicate the time of pH1N1 challenge and immunizations with ChAdOx-NPM1-NA2 and MVA-NPM1-NA2. Significant differences are listed in Table 1. The top of each bar indicates the mean and the line the standard error mean (SEM) **(e–i)**. Each symbol (circle, square and triangles) represents one animal. Asterisks denote significance between indicated groups (*p < 0.05) and were analyzed either by one-way (**h**, **i**) or two-way (**b**–**d**) ANOVA and Bonferroni’s multiple comparisons test when the data were normally distributed or with Kruskal-Wallis and Dunn’s multiple comparisons test when normality was not achieved (**e**–**g**).

Twenty pigs were infected intranasally with 3 × 10^6^ PFU of A/swine/England/1353/2009 (pH1N1). All pigs were successfully infected and shed virus as determined by plaque assays of daily nasal swabs in the first 6 days following pH1N1 inoculation^34^. Four weeks after the pH1N1 inoculation, the pigs were randomly divided into four groups of five animals and were immunized with 5 × 10^8^ IU ChAdOx2-NPM1-NA2 intramuscularly (IM), intranasally (IN) or by aerosol (AE) as previously described^34^. IN delivery was performed with a mucosal atomization device (MAD) generating droplets of ~ 80 to 100 µM diameter delivered in 300 µl volume to restrict the vaccine deposition to the upper respiratory tract. AE delivery by vibrating mesh nebulizer generated droplets of ~ 4.5 µm diameter capable of reaching the entire respiratory tract^35^. Four weeks after the ChAdOx2-NPM1-NA2 immunization the pigs were boosted by the same delivery route with 1.5 × 10^8^ PFU MVA-NPM1-NA2. Unimmunized, but pH1N1 pre-exposed pigs were used as controls (C). The animals were culled four weeks after the boost, and immune responses were evaluated in the bronchoalveolar lavage (BAL), spleen and blood (Fig. 1a).

Circulating antibody responses were measured by ELISA throughout the study, and in BAL at day 83 which is 27 days after final vaccination (Fig. 1b–i). Virus-specific IgG in serum was assayed against live MDCK cells grown pH1N1 and A/swine/Ohio/A01354299/2017 (H3N2) viruses and recombinant NA protein from H3N2 (NA2) (Fig. 1b–d). The IM immunized animals had a significantly higher pH1N1 IgG titer after ChAdOx2-NPM1-NA2 administration (D35 to D49) compared to the other groups. The pH1N1 IgG response decreased 4 weeks post immunization with ChAdOx2-NPM1-NA2 but was boosted by MVA-NPM1-NA2 and remained significantly higher compared to all other groups until D63 and D77, although not reaching the titer after the first immunization (Fig. 1b, Table 1). The second highest serum response was in the AE group on D49 (1:61,440), significantly higher compared to IN and C groups (Table 1). H1N1 neutralization titers were induced by the pH1N1 infection but not boosted by ChAdOx2-NPM1-NA2 and MVA-NPM1-NA2 as previously demonstrated^34^.

**Table 1:** Significant differences between the four groups at the same time-point after immunization.

Assays		Significances identified between groups post immunization								
		Day 35	Day 42	Day 49	Day 56	Day 63	Day70	Day 77	Day 83	Day 87
ELISA IgG (Fig. 1b–d) Two-way ANOVA	H1N1	IM > AE IM > IN IM > C (*P* < 0.0001)	IM > AE IM > IN IM > C (*P* < 0.0001)	IM > AE (*P* = 0.03) IM > IN (*P* < 0.0001) IM > C (*P* < 0.0001) AE > IN (*P* = 0.006) AE > C (*P* = 0.006)	IM > IN IM > C (*P* = 0.006)	IM > AE (*P* = 0.009) IM > IN IM > C (*P* < 0.0001)	Not significant (*P* > 0.05)	IM > IN IM > C (*P* = 0.007)	Not significant (*P* > 0.05)	–
	H3N2	Not significant (*P* > 0.05)	Not significant (*P* > 0.05)	Not significant (*P* > 0.05)	Not significant (*P* > 0.05)	IM > AE (*P* = 0.013) IM > IN (*P* = 0.004) IM > C (*P* = 0.004)	IM > IN (*P* = 0.03) IM > C (*P* = 0.03)	IM > AE (*P* = 0.04) IM > IN (*P* = 0.004) IM > C (*P* = 0.004)	Not significant (*P* > 0.05)	–
	N2	Not significant (*P* > 0.05)	Not significant (*P* > 0.05)	IM > IN IM > C (*P* = 0.003)	Not significant (*P* > 0.05)	IM > IN (*P* = 0.04) IM > C (*P* = 0.0003) AE > C (*P* = 0.04)	IM > IN (*P* = 0.04) IM > C (*P* = 0.0003) AE > C (*P* = 0.04)	IM > IN (*P* = 0.04) IM > C (*P* = 0.0003) AE > C (*P* = 0.04)	IM > C (*P* = 0.003)	–
ELISA IgG (Fig. 6a–c) Two-way ANOVA	H1N1	Not significant (*P* > 0.05)	IM > IN (*P* = 0.008) IM > C (*P* = 0.008)	Not significant (*P* > 0.05)	Not significant (*P* > 0.05)	Not significant (P > 0.05)	IM > C (*P* = 0.03)	Not significant (*P* > 0.05)	Not significant (*P* > 0.05)	Not significant (*P* > 0.05)
	H3N2	IM > C (*P* = 0.048)	IM > C (*P* = 0.028)	IM > C (*P* = 0.02)	Not significant (*P* > 0.05)	Not significant (*P* > 0.05)	IM > C (*P* = 0.04) AE > C (*P* = 0.04)	Not significant (*P* > 0.05)	IM > C (*P* = 0.03)	IM > C (*P* = 0.03)
	N2	Not significant (*P* > 0.05)	IM > AE (*P* = 0.015) IM > C (*P* = 0.004)	IM > C (*P* = 0.002)	IM > AE (*P* = 0.002) IM > IN (*P* = 0.047) IM > C (*P* = 0.003)	IM > AE (*P* = 0.005) IM > IN (*P* = 0.005) IM > C (*P* = 0.005)	IM > AE (*P* = 0.006) IM > IN (*P* = 0.005) IM > C (*P* = 0.005)	IM > AE (*P* = 0.019) IM > IN (*P* = 0.018) IM > C (*P* = 0.017)	IM > AE (*P* = 0.0007) IM > IN (*P* = 0.004) IM > C (*P* = 0.001)	IM > C (*P* = 0.003)

Although serum H3N2 IgG responses were highest in the IM and AE groups, these were not significantly different from IN and C groups following the first immunization (Fig. 1c). However, a week after boosting with MVA-NPM1-NA2 (D63) the IM group had a significantly higher response (1:36,864) in comparison to the other groups, which declined over time but remained significantly different from IN and C at D70 and D77 (Table 1). IM immunization significantly boosted IgG responses to recombinant NA2 protein on D49 compared to IN and C groups (Table 1). The NA2 response peaked a week after the MVA boost (D63), remained high until D70 (1:114,688) and declined from D77 onwards and was still significantly higher than the C group at D83 (Table 1). The IgG NA2 specific response was also increased in the AE group compared to C. No significant differences were observed in serum pH1N1 and H3N2 specific IgA responses between the groups (data not shown). Intranasal immunization did not boost significantly pH1N1, H3N2 or NA2 specific serum responses. There was no increase in pH1N1 neutralizing activity in the sera of any of the immunized groups compared to controls, suggesting that the vaccines did not boost the response to either H1 or N1, as these are the targets for neutralization. We were unable to detect neutralizing activity against H3N2 in serum from the ChAdOx2-NPM1-NA2 and MVA NPM1-NA2 pigs immunized by any route.

No significant differences of pH1N1 specific IgG and IgA responses were observed in BAL at D83 between groups (Fig. 1e–i). In contrast AE immunization induced a significantly greater H3N2 specific IgG response in BAL compared to IN and C groups (Fig. 1f) and a trend for higher H3N2 IgA (Fig. 1i, *p* = 0.09, one-way ANOVA and Bonferroni’s multiple comparisons test).

In summary, after pH1N1 pre-exposure, IM prime boost immunization with ChAdOx2-NPM1-NA2 and MVA-NPM1-NA2 induced high serum IgG titers against both pH1N1 and H3N2, while AE delivery induced high anti-H3N2 IgG and IgA titers in BAL in agreement with our previous study with ChAdOx2-NPM1-NA2^34^.

### Cytokine responses following prime boost immunization in pH1N1 pre-exposed pigs

**Fig. 2:**
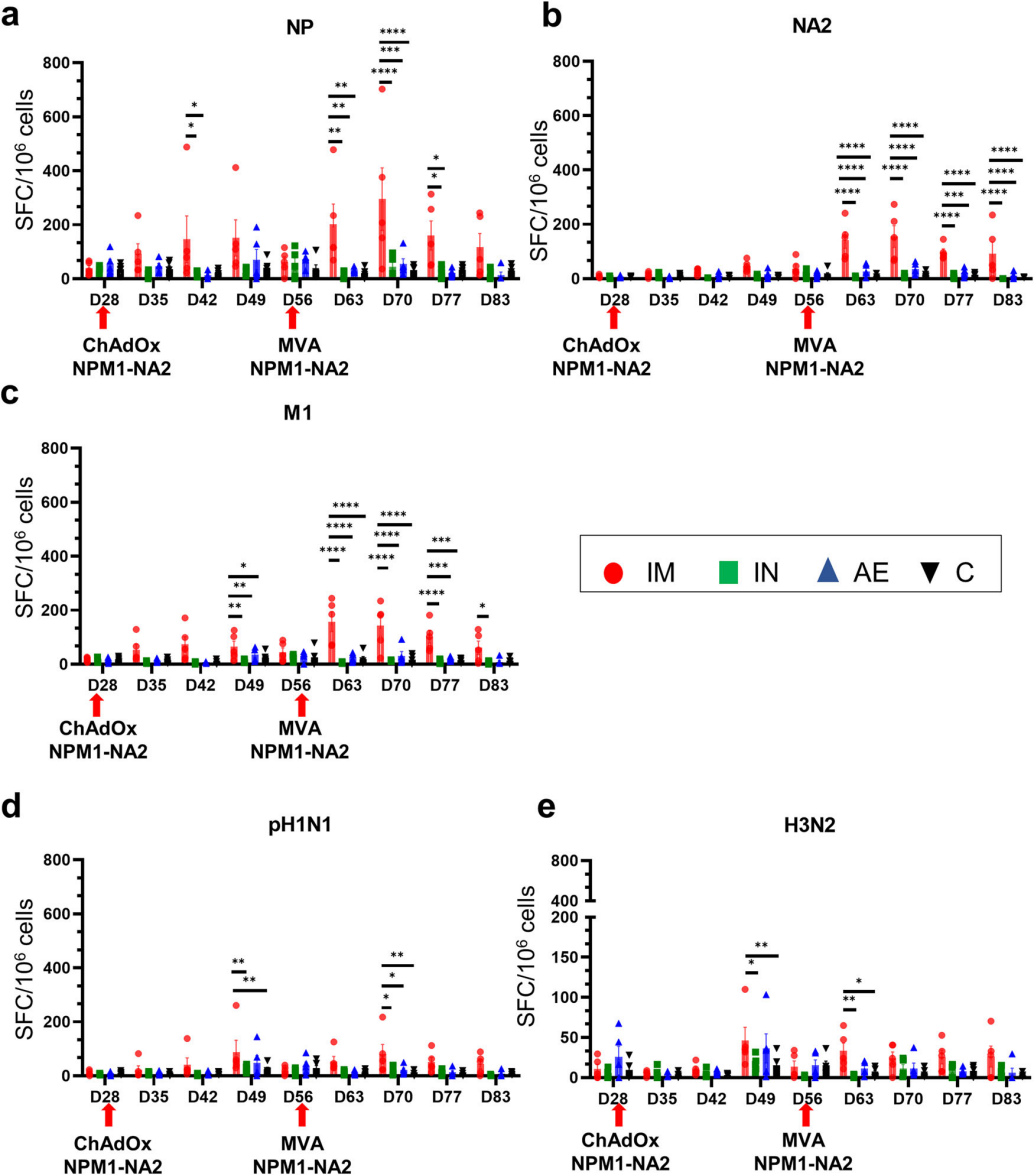
IFNγ ELISpot responses in PBMC. IFNγ secreting spot forming cells (SFC) were enumerated following stimulation with a pool of peptides covering NP (**a**), NA2 (**b**) and M1 (**c**) proteins or pH1N1 (**d**) and H3N2 (**e**) viruses. The arrows below D28 and D56 indicate the immunizations with ChAdOx-NPM1-NA2 and MVA-NPM1-NA2. Each symbol represents an individual animal, the top of the bar the mean and the line the standard error (SEM). Asterisks denote significance between indicated groups (*p < 0.05, **p < 0.01, ***p < 0.001, ****p < 0.0001). The data were analyzed by two-way ANOVA and Bonferroni’s multiple comparisons test.

**Fig. 3:**
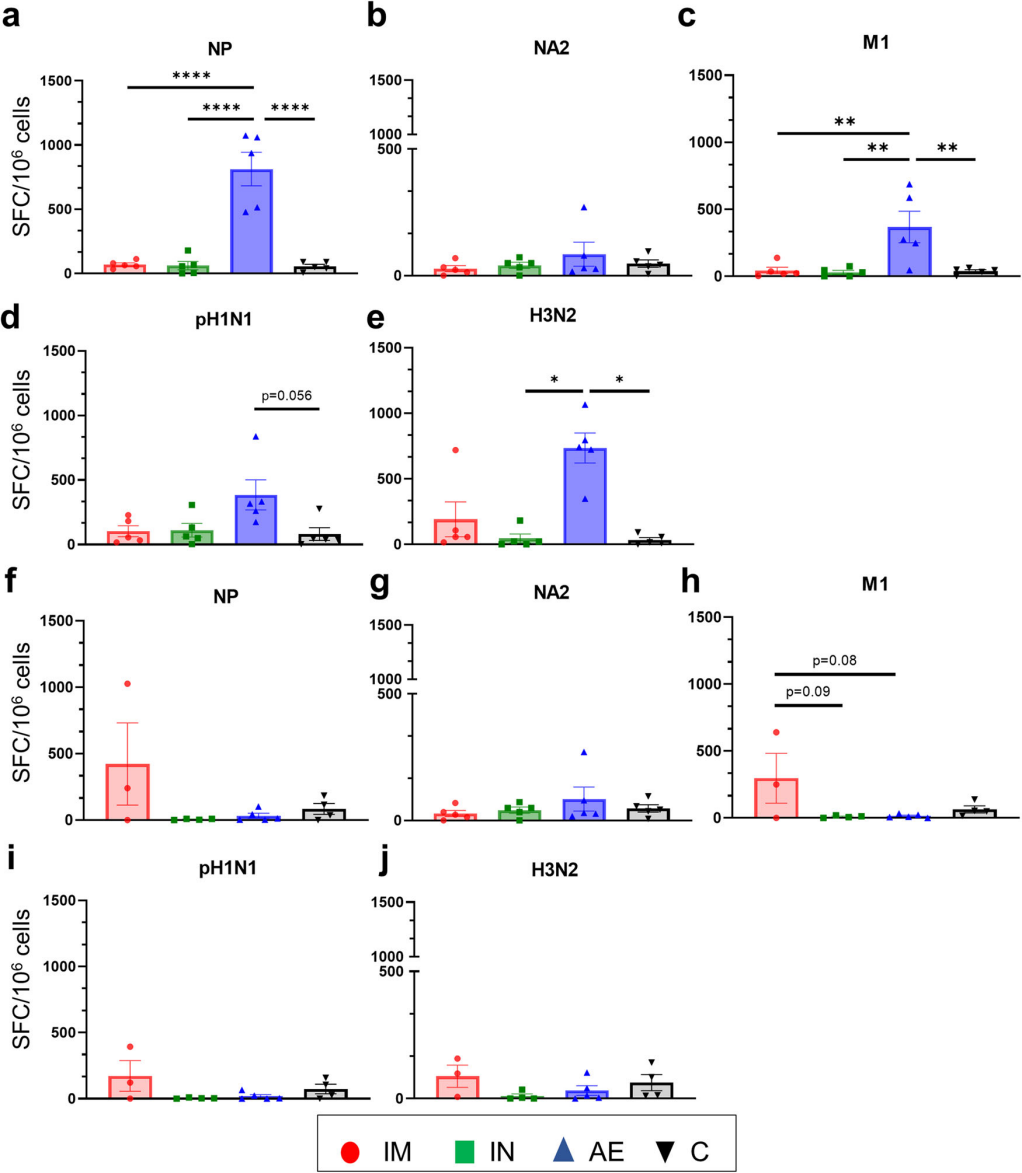
IFNγ ELISpot responses in BAL and spleen. IFNγ secreting spot forming cells (SFC) were enumerated in BAL (**a**–**e**) and spleen (**f**–**j**) on D83. Cells were stimulated with a pool of peptides covering NP (**a**, **f**), NA2 (**b**, **g**) and M1 (**c**) proteins or pH1N1 (**d**, **i**) and H3N2 (**e**, **j**) viruses. Each symbol represents an individual animal, the top of the bar the mean and the line the standard error (SEM). Asterisks denote significance between indicated groups (*p < 0.05, **p < 0.01, ****p < 0.0001) and were analyzed either by one-way ANOVA and the Bonferroni multiple comparisons test when the data were normally distributed (**a–c**, **f**, **h**, **i**) or with Kruskal–Wallis and Dunn**’**s multiple comparisons test when normality was not achieved (**d**, **e**, **g**, **j**).

IFNγ ELISpot analysis was performed to quantify IFNγ producing cells in peripheral blood mononuclear cells (PBMC, Fig. 2), BAL (Fig. 3a–e) and spleen (Fig. 3f–j) following stimulation with either pH1N1 and H3N2 live viruses or with pool of overlapping peptides covering the NP, M1 and NA2 proteins included in the vaccine (Figs. 2 and 3). IM immunization with ChAdOx2-NPM1-NA2 significantly increased the NP-specific response in PBMC after D42 and M1, pH1N1 and H3N2 responses on D49 (Fig. 2a, c, d, e). IM boost with MVA-NPM1-NA2, induced the greatest response to NP, M1 and NA2 which remained higher compared to the other groups until the end of the study D83. Significant differences in response between groups were reached at different time points after prime boost immunizations as indicated in Fig. 2.

As with the antibody responses, cellular responses in the BAL were highest following AE immunization (Fig. 3a–e). AE induced the highest responses to NP, M1 and H3N2 stimulation and the response to pH1N1 was higher than any other although not significantly different (*p* = 0.056 relative to the control group, Kruskal–Wallis and Dunn’s multiple comparisons test). The IFNγ ELISpot responses in the spleen (Fig. 3f–j) did not reveal significant differences between the groups, although a trend was observed to M1 stimulation (only 3 spleen samples were available for the IM group) (Fig. 3h).

**Fig. 4:**
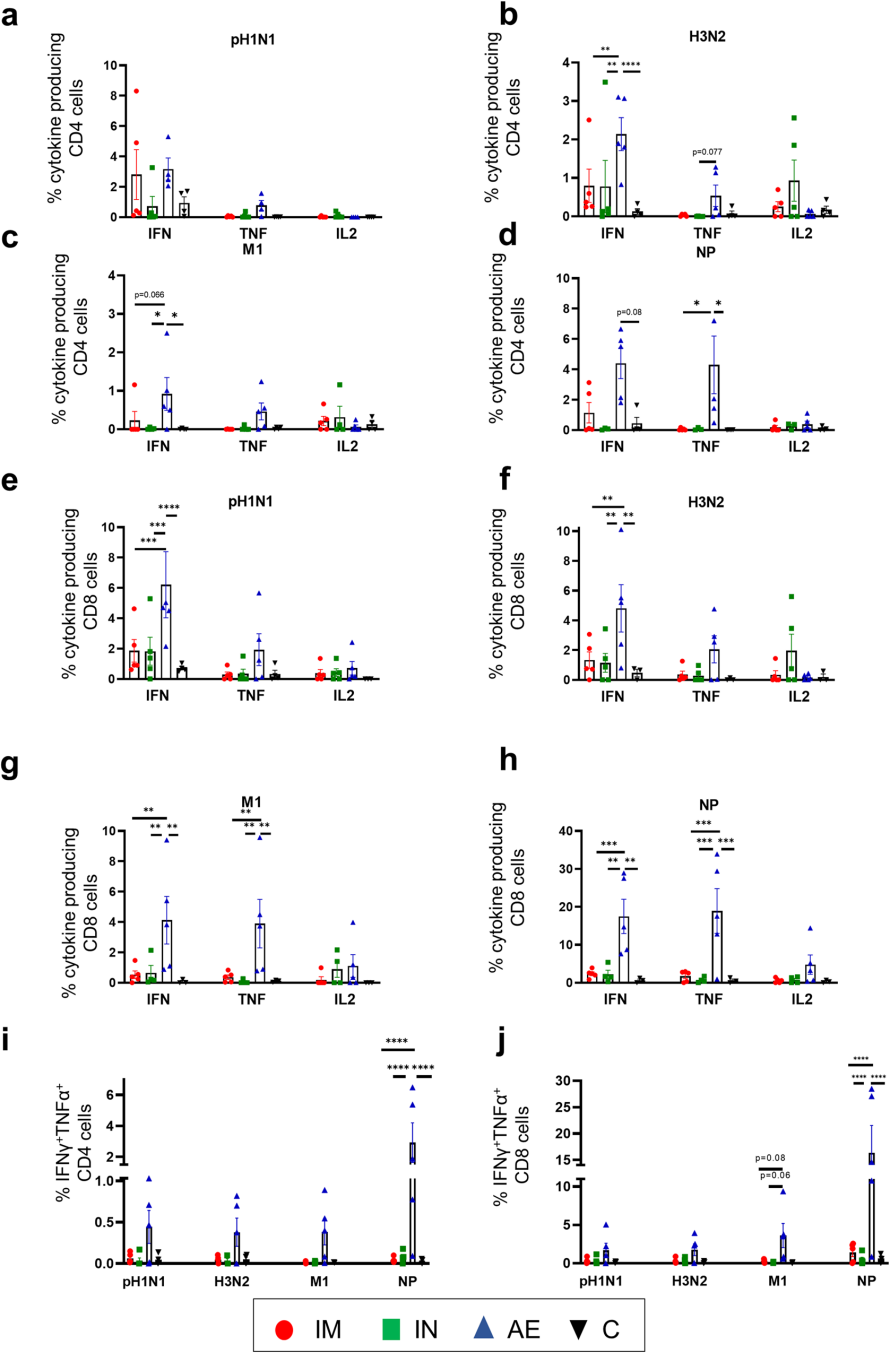
T cell cytokine responses in BAL. BAL was collected four weeks after the MVA-NPM1-NA2 immunization. Cryopreserved cells from D83 were thawed, stimulated with pH1N1, H3N2, NP or M1 and IFNγ, IL-2, TNF and IFNγTNF cytokine secretion was measured in CD4 (**a**–**d**, **i**) and CD8 (**e**–**h**, **j**). T cells by intracellular cytokine staining. Each symbol represents an individual animal, the top of the bar the mean and the line the standard error (SEM). Two-way ANOVA and Bonferroni’s multiple comparisons test were used to compare responses between groups and asterisks indicate significant differences (*p < 0.05, **p < 0.01, ***p < 0.001, ****p < 0.0001).

T cell responses were also analyzed by intracellular cytokine staining (ICS) in BAL (Fig. 4). IFNγ, TNF, IL-2 and IFN/TNF production by CD4 and CD8β T cells was measured following pH1N1, H3N2, NP and M1 stimulation (gating strategy shown in Supplementary Fig. 1). AE immunization was the only regimen that induced statistically higher responses compared to the other groups. AE immunization induced the highest IFNγ and TNF producing H3N2 and NP-specific CD4 cells in BAL (Fig. 4b, d). The CD8β T cells exhibited the highest IFNγ production to all stimuli with NP specific CD8β T cells having the highest frequencies of IFNγ (17.48%) and TNF (18.91%). AE immunization also induced the highest M1 and NP-specific TNF CD8β producing T cells (Fig. 4g, h) and NP-specific IFNγ/TNF co-producing CD4 and CD8β T cells (Fig. 4i, j). IL-2 frequencies were lower and did not show any differences between the groups.

These data indicate that IM prime boost immunization induced a strong antibody and cellular IFNγ response in PBMC, while AE induced the highest antibody and T-cell responses in the BAL with very high frequency of cytokine producing CD8 T cells.

### Efficacy of prime boost immunization in pH1N1 pre-exposed pigs against H3N2 challenge

Following the immunogenicity study showing high systemic and local immune responses after different routes of prime and boost with ChAdOx2-NPM1-NA2 and MVA-NPM1-NA2 in pH1N1 pre-exposed animals, we next investigated the efficacy of these immunization regimens in protecting against H3N2 influenza infection (Supplementary Fig. 2a). Twenty-four animals were inoculated with 3 × 10^6^ PFU pH1N1 and as with previous studies, virus shedding was detected in nasal swabs taken daily for the first 6 days to confirm the successful infection of all animals (Supplementary Fig. 2b). Four weeks later the pigs were randomly divided into four groups of six animals and immunized either IM, IN or by AE with ChAdOx2-NPM1-NA2 and MVA-NPM1-NA2 4 weeks apart as described above. Four weeks after the MVA-NPM1-NA2 boost, all animals were infected intranasally with 9 × 10^7^ pfu of A/swine/Ohio/A01354299/2017 (H3N2). Animals were humanely euthanized four days later. This time point was chosen as it allows four days for monitoring virus shedding in daily nasal swabs, there is still significant viral load in the lungs and BAL and lung pathology is well developed^30,31,36^. Three animals reached their humane end point before the completion of the study due to bacterial infection unrelated to the procedures. Thus, the IN and C groups contained only four and five animals, respectively.

**Fig. 5:**
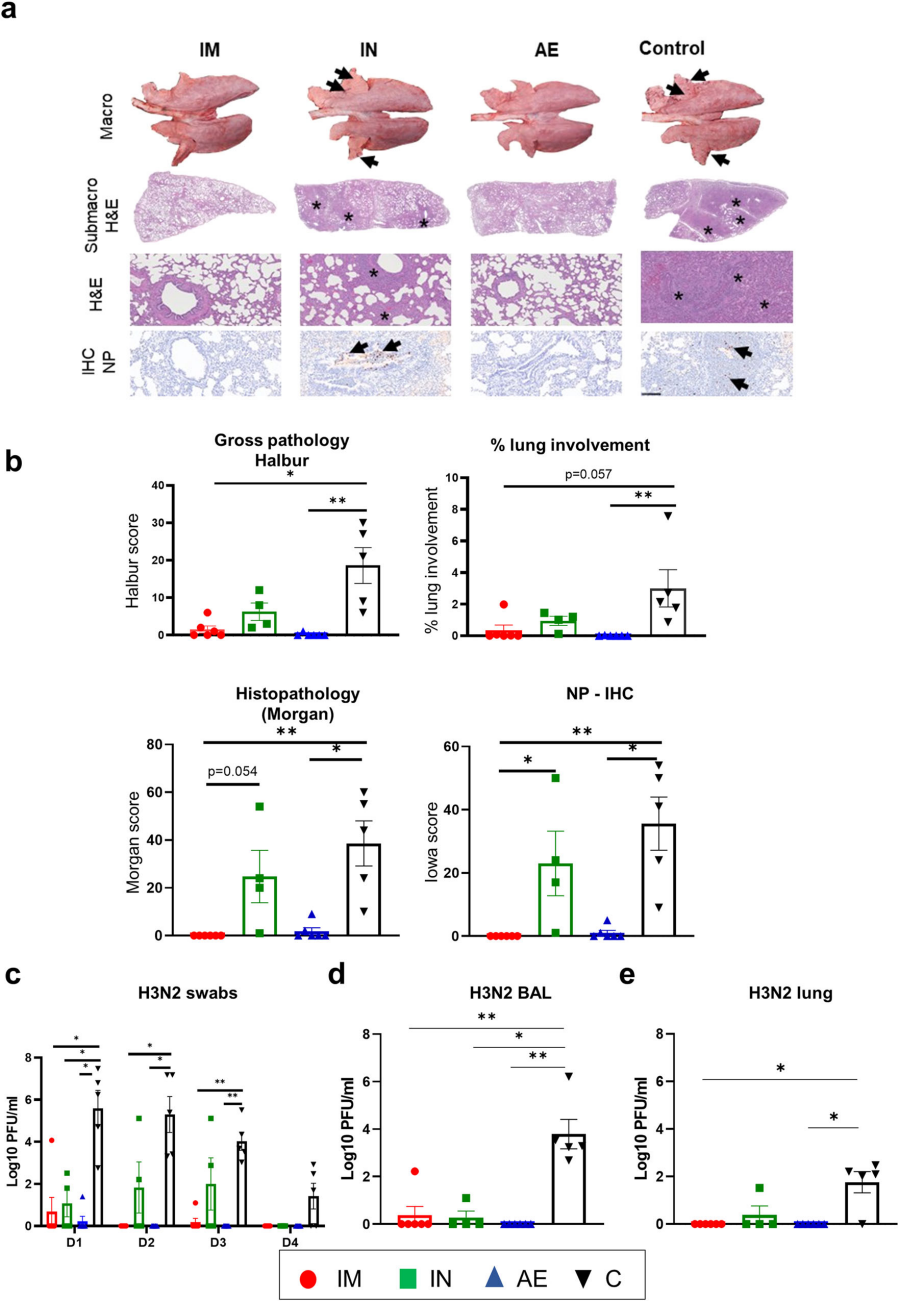
Lung pathology and viral load following re-challenge with H3N2. Representative lung gross pathology, histopathology (submacroscopic and microscopic) and immunohistochemical NP staining of each group. Gross pathology is observed as areas of consolidation (arrows). At submicroscopic histopathology, areas of bronchopneumonia are characterized by the obliteration of the normal airway structures, with inflammatory cell infiltration (*), that can be observed at microscopic level as bronchiolo- and broncho-interstitial pneumonia with necrosis of epithelial cells and inflammatory infiltrates in the airways and parenchyma (*). Virus NP is detected by IHC (brown stain, arrows) within the bronchiolar wall and luminae and occasionally within the parenchyma (**a**). Gross (top graphs) and histopathology scores are shown, including the percentage of lung surface with lesions, lesion scores and histopathology scores (**b**). Virus load was determined by plaque assay in daily nasal swabs (**c**) post H3N2 infection (D1-D4), in the BAL (**d**) and lung (**e**) 4 days post infection (D87). The top of each bar indicates the mean and the line the SEM. Each symbol (circle, square and triangles) represents one animal. Asterisks denote significance between indicated groups (*p < 0.05, **p < 0.01) and were analyzed either by one-way ANOVA and Bonferroni’s multiple comparisons test when the data were normally distributed (**c**) or with Kruskal–Wallis and Dunn’s multiple comparisons test when normality was not achieved (**b**, **d**, **e**). Bar = 100 micrometers.

Representative lung gross pathology, histopathology (submacro- and microscopic) and immunohistochemical NP staining are shown (Fig. 5a). Gross pathology was observed as areas of consolidation in the cranial and medial lobes. The unimmunized group (C) had a significantly higher gross pathology score in comparison to the IM and AE, but not the IN group. Histopathological analysis showed areas of bronchopneumonia characterized by the obliteration of the normal airway structures with inflammatory cell infiltration, alveolar exudation, and bronchiolar and broncho-interstitial pneumonia with necrosis of epithelial cells and inflammatory infiltrates in the airways and parenchyma present in the C and IN groups, while the IM and AE groups did not show histopathological lesions. Labeling of influenza NP by immunohistochemistry (NP-IHC) was seen within the epithelial cells and exudates and occasionally within the parenchyma of unimmunized and intranasally immunized animals. NP labeling was not detected in the IM group and was only detected in one animal of the AE group (Fig. 5b).

The IM and AE groups showed the greatest reduction in virus shedding in daily nasal swabs, while the IN and C groups shed virus consistently until day 3 and 4 respectively (Fig. 5c). One animal in the AE group showed a low titer of virus at day 1 only and one animal from the IM group a low titer on 1- and 3-days post infection (dpi). No virus was detected in the BAL and lung of the AE immunized animals at 4 dpi, while in the IM and IN groups one animal had low levels of virus in BAL and one from the IN group in lung at 4 dpi (Fig. 5d, e). There was reduced pathology and virus load in the IN group, but the only parameter that differed significantly from the controls was virus load in BAL at 4 dpi.

These results indicate that IM and AE immunization abolished lung pathology and were highly efficient in controlling virus shedding and viral load in lung.

### Immune responses after H3N2 challenge

**Fig. 6:**
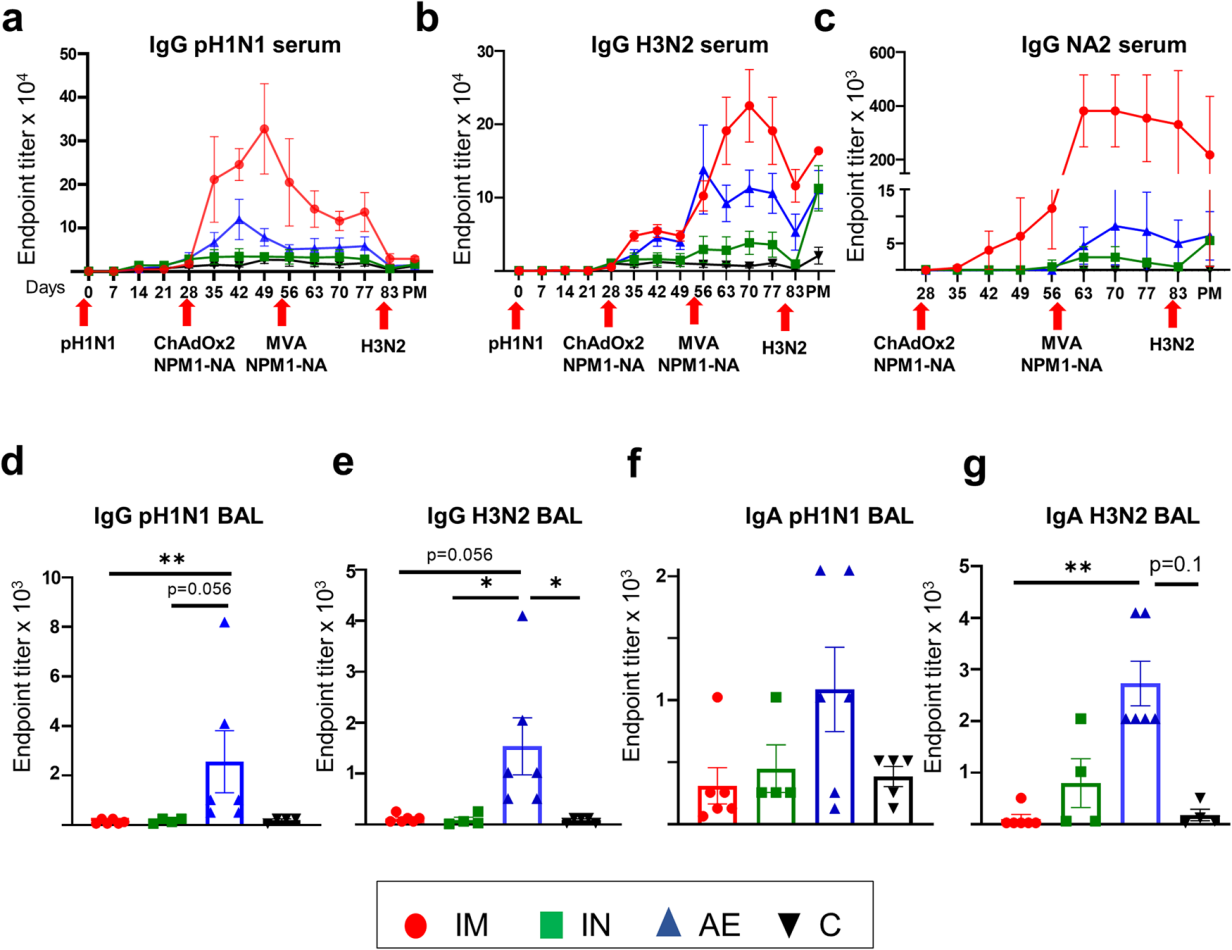
Systemic and mucosal antibody responses. pH1N1 (**a**), H3N2 (**b**) and NA2 (**c**) specific IgG responses in serum were determined by ELISA at the indicated time points. pH1N1 (**d**) and H3N2 (**e**) IgG and H1N1 (**f**) and H3N2 (**g**) IgA responses in BAL, were determined by ELISA 4 days after the H3N2 challenge. The mean and standard error (SEM) is presented in each time point (**a**–**c**). Significant statistical differences are listed in Table 1. The top of each bar indicates the mean and the line the SEM. Each symbol (circle, square and triangles) represents one animal (**d**–**g**). Asterisks denote significance between indicated groups (*p < 0.05, **p < 0.01) and were analyzed either by one-way (**g**) or two-way (**a**–**c**) ANOVA and Bonferroni**’**s multiple comparisons test when the data were normally distributed or with Kruskal-Wallis and Dunn’s multiple comparisons test when normality was not achieved (**d–f**).

IM immunization induced the highest IgG response to pH1N1, H3N2 and NA2 in serum in agreement with the first immunogenicity study described above (Fig. 6a–c and Table 1). Four days following H3N2 infection (D87), serum H3N2 specific IgG titers increased in all groups with IM having the greatest titer compared to C group. AE immunization induced the highest pH1N1 and H3N2 specific IgG and IgA in BAL 4 days post H3N2 infection (D87) (Fig. 6d–g).

**Fig. 7:**
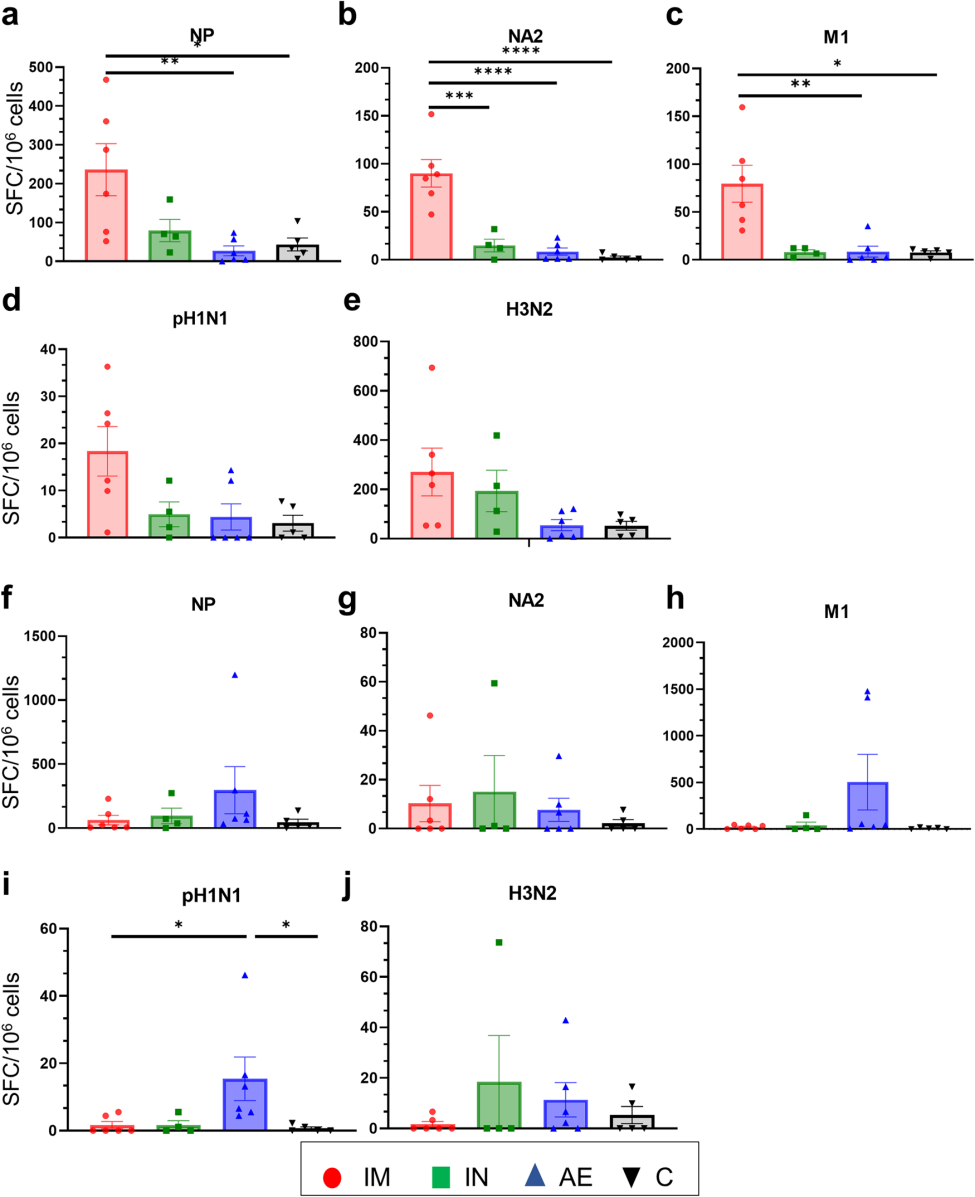
IFNγ ELISpot responses in PBMC and BAL. IFNγ secreting spot forming cells (SFC) were enumerated in PBMC (**a**–**e**) and BAL (**f**–**j**) on D87. Cells were stimulated with a pool of peptides covering NP (**a**, **f**), NA2 (**b**, **g**) and M1 (**c**, **h**) proteins or pH1N1 (**d**, **i**) and H3N2 (**e**, **j**) viruses. Each symbol represents an individual animal, the top of the bar the mean and the line the standard error (SEM). Asterisks denote significance between indicated groups (*p < 0.05, **p < 0.01, ***p < 0.001 ****p < 0.0001) and were analyzed either by one-way ANOVA and the Bonferroni multiple comparisons test when the data were normally distributed (**a**, **b**, **c**, **e**) or with Kruskal–Wallis and Dunn’s multiple comparisons test when normality was not achieved (**d**, **f**–**j**).

The cellular immune responses in PBMC and BAL were measured by IFNγ ELISpot (Fig. 7). The IM immunized group had the highest number of NP-, NA2- and M1- specific IFNγ producing cells in PBMC (Fig. 7a–c). The IFNγ responses in BAL did not differ between the groups four days after H3N2 infection, except for the response to pH1N1 in the AE group (Fig. 7i). Similarly, the highest antigen specific CD8 responses in the BAL were detected in the AE group by intracytoplasmic staining (Supplementary Fig. 3). The very low cytokine responses after H3N2 challenge compared to the responses before challenge (albeit in a different experiment) might be due to activation induced cell death of cells which have been previously activated in vivo by the challenge H3N2 virus. There was no obvious influx of memory/effectors cells from the systemic circulation to the BAL in the IM group.

Taken together, these data indicate that the IM immunization elicited high Ab and T-cell responses in the periphery, while AE immunization induced high responses in the BAL and this difference was maintained after the H3N2 challenge.

## Discussion

Cross protective T cell immune responses against conserved internal influenza A virus antigens such as NP and M1, have been associated with reduced virus shedding and limited severity of disease following influenza infection in humans^16–19^. This evidence, combined with a long history of animal studies demonstrating the protective effect of T cells induced by influenza virus infection, is the rationale for developing a BPIV that induces a strong T cell response against conserved internal antigens^37^. More recently, antibody responses to NA were also shown to provide broader protection^29,38^. Since almost all human influenza immunizations are given to influenza-exposed rather than influenza-naïve humans, to model this we exposed pigs to pH1N1 prior to vaccination rather than immunizing influenza-naïve pigs. We demonstrate that following pH1N1 exposure, intramuscular or aerosol prime boost regimens with ChAdOx2-NPM1-NA2 and MVA NPM1-NA2, significantly reduced lung pathology, virus shedding and lung viral load after H3N2 influenza challenge. In contrast intranasal immunization, restricted to the upper respiratory tract, had a very limited effect on virus load and pathology in this model.

We have demonstrated in young and adult humans that a two dose IM heterologous prime boost regimen with ChAdOx1 NP + M1 and MVA-NP + M1 given either 8 or 52 weeks apart, significantly increased the proportion of cross-reactive T cells and maintained them at high frequencies for 18 months after immunization^21^. We further showed that inclusion of a third antigen, the HA, in ChAdOx-NPM1-HA and MVA-NPM1-HA significantly reduced virus shedding in pigs after prime boost vaccination against homologous H1N1pdm09 virus challenge^39^. Here we show that inclusion of the NA, in ChAdOx-NPM1-NA2 and MVA NPM1-NA2 induced powerful T cell and antibody responses in pH1N1 pre-exposed pigs and protected them against H3N2 challenge.

The intramuscular and aerosol prime boost immunizations with ChAdOx-NPM1-NA2/ MVA-NPM1-NA2 generated distinct cellular and humoral responses. Local T and antibody lung responses were only observed after aerosol delivery targeting the lower and upper respiratory tract. In contrast, intramuscular administration boosted T cell and antibody responses in blood but had a weak effect on the lung mucosal response measured in BAL after pH1N1 pre-exposure. As the vaccines contain three immunogens, NP, M1 and the NA envelope antigen it is difficult to dissect the contribution of each antigen or arm of the immune response to protection. Furthermore, this may differ according to the route of immunization. In the IM group the most likely mechanism of protection may be the high titre of anti-NA2 antibodies especially as the immunizing NA is matched to the challenge H3N2 virus. Although we could not detect neutralizing activity in the sera of these pigs, other Fc mediated mechanisms may contribute to the killing of infected cells^40^. The IM regimen also induced a very strong systemic T cell response; however, we have no evidence for recruitment of these cells to the site of infection in the respiratory tract, which may reflect the sensitivity of the assay used or that there is a real difference in the effects of these immunization regimes.

In contrast, AE immunization induced detectable IgG and IgA antibody against H3N2 in the BAL. The serum anti-NA2 titers was lower compared to the IM group, but still significantly higher compared to the control. There was a much stronger local BAL T cell response in the AE group. We speculate that both T-cell and antibody responses may contribute to the protection seen in this group. Aerosol immunization with another T cell candidate vaccine, S-FLU, which contains all viral proteins induced a powerful T cell response (but not neutralizing antibody response) and reduced pathology, but did not prevent virus replication or shedding, although in these experiments, there was no pH1N1 pre-exposure^30,36^. We speculate that T-cell responses can reduce pathology in severe disease, but combined T-cell and antibody responses are required to prevent pathology and virus shedding, which will also prevent onward transmission^39,41^. It should also be noted that AE immunization delivers only a third of the dose so that this route appears to be extremely efficient in inducing immune responses^9,35^.

A limitation of this study is that we have not demonstrated broad immunity because the challenge virus was matched to the NA in the vaccine. That question could be addressed by challenging with a virus with a different NA sequence. However NA exhibits a slower drift that is discordant with that of HA and antibody responses against NA typically show broader cross-reactivity^42–44^. Further studies with vaccines containing only NPM1 or NA2 will allow us to determine whether antibodies against viral envelope proteins are essential for prevention of virus shedding and whether mismatch of immunizing and challenge NA reduces protection in pigs as has been demonstrated in small animals^29,41,45^. These experiments will also reveal whether T cells directed solely against internal proteins are sufficient to reduce pathology and severe disease in a large natural host animal. In addition, further studies should investigate whether the more clinically deployable regimens of priming and boosting with the same viral vector or using a single immunization with ChAdOx2 provides similar protection. A final crucial issue is the duration of protection, whether antibody or T cell mediated. Even if protection is relatively short lived, a BPIV will be highly advantageous because it could be used without changing it annually or in the face of a pandemic. Lessons learned from these influenza studies may be relevant for SARS-CoV-2 and other respiratory viruses.

## Methods

### ChAdOx2-NPM1-NA2 and MVA-NPM1-NA2 viral vectored vaccines

ChAdOx2 is a replication deficient (E1 and E3 deleted) simian adenovirus^46^, which was engineered to express swine Influenza A virus nucleoprotein (NP) and matrix 1 (M1) as a fusion protein (NPM1) and NA2 separated from NPM1 by a 2A ribosome skipping sequence. The NP and M1 protein ORFs were derived from A/swine/England/1353/2009 (GenBank accession number KR701098 and KR701100). The neuraminidase (NA2) is from H3N2 strain A/swine/Ohio/A01354299/2017 (GenBank accession number MF801571)^34^. MVA is a replication incompetent poxvirus vector engineered to express the same NPM1 fusion protein under the control of the F11 promoter and the same NA2 under the control of the modified H5 promoter^47^.

### Immunization and challenge animal studies

Animal studies were approved by the ethical review processes at the Animal and Plant Health Agency (APHA) and the Pirbright Institute (study numbers PP9878849-2-001 and P47CE0FF2-1-015) in accordance with the UK Government Animal (Scientific Procedures) Act 1986.

For the immunogenicity study, twenty 6-week-old female Landrace x Large White pigs were obtained from a commercial high-health status herd (average weight of 9.6 kg at the beginning of the study). Pigs were screened for the absence of influenza A virus antibody reactivity by HAI with four swine Influenza A virus antigens from H1N1pdm09, H1N2, H3N2 and avian-like H1N1 lineages and serum IgG titres against pH1N1 and H3N2 were assayed by ELISA. All pigs were inoculated intranasally with 3 × 10^6^ PFU of A/swine/England/1353/2009 (pH1N1) MDCK grown virus in a total of 4 ml (2 ml per nostril) using a mucosal atomization device (MAD, Wolfe-Tory Medical) (Fig. 1a). The inoculation dose for pH1N1 was in agreement with previous studies, resulting in consistent reproducible infection, virus shedding, lung vial load and lung pathology^34,36,48^. Following pH1N1 challenge, daily nasal swabs were collected for 7 days to assess virus load by plaque assays and blood samples were collected weekly for PBMC isolation, as previously described^30^. Briefly, the nasal swabs were collected in 2 ml of virus transport medium, containing tissue culture medium 199 (Sigma-Aldrich, Cat. No: M0650) and supplemented with 25 mM HEPES, 0.035% sodium bicarbonate, 0.5% BSA, penicillin, streptomycin, and nystatin. For the viral titration with plaque assays on MDCK cells, the nasal swabs samples were 10-fold serially diluted in Dulbecco’s Modified Eagle’s Medium (DMEM, Gibco™) and 200 µl were overlayered on confluent MDCK cells in 12-well tissue culture plates. One hour later the plates were washed and 1 ml of % agarose/medium (1:3) was added. After 72 h of incubation at 37 °C and 5% CO2 the plaques were visualized using 0.1% crystal violet. For the PBMC isolation blood was first 1:1 diluted in PBS and then subjected to a 30 min at 920 × *g* centrifugation on density gradient medium (Biolegend). PBMCs were then harvested, washed twice with PBS and once with culture media and then were cryopreserved in FCS with 10% DMSO (Sigma-Aldrich, D2650). Four weeks post pH1N1 infection, the animals were randomly assigned tο four groups and immunized with 5 × 10^8^ infectious units (IU) ChAdOx2-NPM1-NA2 as follows: 1) intramuscularly (IM) with 1 ml administered in each trapezius muscle behind the ear); 2) intranasally (IN) with 300 µl per nostril administered with a MAD, with the aim of restricting the vaccine to the upper respiratory tract;^34,35^ 3) aerosol (AE) with 1 ml administered over 2–5 min using an Aerogen Solo vibrating mesh nebulizer (Aerogen, Dangan, Galway, Ireland), and 4) unimmunized controls. The animals in the IN and AE groups were anaesthetized prior to immunization with a mixture of 5 mg/kg Zoletil (2.5 mg/kg of Tiletamine + 2.5 mg/kg of Zolazepam) and 0.05 mg/kg Domitor (medetomidine). After four weeks, the animals were boosted with 1.5 × 10^8^ PFU of MVA-NPM1-NA2 by the same immunization route as they were primed. Four weeks later the animals were humanely killed and blood, bronchoalveolar lavage (BAL), lung and spleen were collected.

For the efficacy study twenty-four 6-weeks old female influenza virus free pigs were used (average weight 9.6 kg) and inoculated intranasally with 3 × 10^6^ PFU pH1N1 as above (Supplementary Fig. 2a). Four weeks later the animals were randomly divided into groups and primed with 5 × 10^8^ IU ChAdOx2-NPM1-NA2 either IM, IN or AE. The animals were boosted 4 weeks later with 1.5 × 10^8^ PFU of MVA-NPM1-NA2 using the same immunization route. Two pigs assigned to the IN and C group, reached their humane endpoints 14 days post pH1N1 inoculation, which was 2 weeks prior to the first immunization, due to osteomyelitis of bacterial origin. A third pig was euthanized 2 days after ChAdOx2 IN immunization, because of bacterial polyserositis, unrelated to the vaccination. Thus, the IN and C groups were left with four and five animals, respectively. Although we were not able to identify the aetiology of the intercurrent infection that caused the 3 pigs to reach their human endpoints, as a precaution we administered a single dose of 20 mg/kg Engemycin 10% (oxytetracycline) to all pigs.

Four weeks after the boost with MVA-NPM1-NA, all animals were intranasally infected with 9 × 10^7^ pfu of A/swine/Ohio/A01354299/2017 (H3N2) MDCK grown virus by administration of 2 ml to each nostril using a MAD300 device. The H3N2 inoculation dose was chosen based on pilot studies and was also higher because the pigs were older and heavier by that time (average weight 66.7 kg). Pigs were culled four days later for assessment of pathology, virus load and immune responses. For logistical reasons, two H3N2 challenges were performed, with half of the animals challenged at 28 days post boost and the remainder at 30 days post boost. As the analysis of samples from pigs challenged at days 28 and 30 did not show any significant differences, for simplicity in presentation, the results of the assays carried out on pigs challenged on both days have been amalgamated in all figures. At postmortem blood, BAL and lung were collected and processed as described before^30,31^. Daily nasal swabs were collected after the H3N2 challenge for assessment of virus shedding by plaque assays, as described above. Viral load was also assessed in BAL and lung by plaque assays post H3N2 infection^30,31^.

### Lung pathology and immunohistochemistry

Gross and histopathological analyses were performed as previously described^36^. Briefly, the lungs were removed, and digital photographs were taken of the dorsal and ventral aspects. Macroscopic pathology was scored blind as previously reported^49^. The percentage of the lung displaying gross lesions for each animal was calculated using image analysis software (Nikon NIS-Ar) on the digital photographs. Lung tissue samples from cranial, middle, and caudal lung lobes were taken from the right lung and collected into 10% neutral-buffered formalin for routine histological processing. Formalin-fixed tissues were paraffin wax embedded, and 4-mm sections were cut and routinely stained with H&E^30^. Immunohistochemical detection of influenza A virus NP was performed in 4-mm tissue sections. Histopathological changes in the stained lung tissue sections were scored by a veterinary pathologist blinded to the treatment group. Lung histopathology was scored using five parameters (necrosis of the bronchiolar epithelium, airway inflammation, perivascular/bronchiolar cuffing, alveolar exudates, and septal inflammation) on a five-point scale of 0–4 and then summed to give a total slide score ranging from 0–20 per lobe (∼1.5 × 3 × 1.5 cm^2^). and a total animal score from 0 to 60^31^. A mean score for the three lung lobes was calculated for each animal. The individual lung lobes were also scored using the “Iowa” method, which takes into account the amount of viral Ag present in the sample^50^.

### ELISA

Endpoint titer ELISAs for pH1N1, H3N2 and recombinant NA protein from H3N2 A/swine/Ohio/A01354299/2017 (NA2) (sequence matched to the vaccine antigen, Genbank accession number: ATE49827, produced by The Native Antigen company) were performed for serum and BAL as described before^34^. Briefly, 96-well microtiter plates (Nunc MaxiSorp, Sigma Aldrich, UK) were overnight coated either with 1 μg/ml of recombinant NA protein (N2) (sequence matched to the vaccine antigen, GenBank accession number: ATE49827, and produced by The Native Antigen Company) or with live pH1N1 or H3N2 viruses grown in MDCK cells (1 × 10^5^ PFU per well). Two hundred microliters blocking solution of 4% (w/v) milk powder (Marvel) in PBS, supplemented with 0.05% Tween-20 (PBS-T) was used, and the plates were incubated for 2 h at room temperature. The starting dilutions for serum and BAL were 1:20 and 1:2, respectively, and the plates were incubated on a rocking platform. One hour later, the plates were washed 3 times with PBS-T and 100 μl of horseradish peroxidase (HRP)-conjugated goat anti-pig IgG (Bio-Rad, Cat. No.: AAI41P), diluted in PBS-T with 4% milk powder was added for 1 h. The plates were washed 4 times with PBS-T and were developed with 100 μl of 3,3′,5,5′-tetramethylbenzidine (TMB) High Sensitivity substrate solution (BioLegend, London UK). The reaction was stopped with 100 μl 1 M H_2_SO_4_ and the plates were read with the Cytation3 Imaging Reader (Biotek, Swindon, UK). The quantities of antibody were determined as the reciprocal value of the dilution that gave the first reading above the cut-off value (end-point titer). Cut-off values were determined as mean blank ODs plus 2-fold standard deviations.

### IFNγ ELISpots and intracellular cytokine staining

Cryopreserved cells from BAL, spleen and PBMC were used to assess the frequency of IFNγ-producing cells by ELISpot as described before^34^. Briefly, MultiScreen-HA ELISPOT plates (Merck Millipore) were coated with 0.5 µg/ml anti-pig IFNγ (clone P2G10; BD Pharmingen) overnight. The plates were washed with PBS and blocked with culture media [RPMI 1640 with stable glutamine supplemented with 10% fetal calf serum (FCS), 100 IU/ml penicillin, and 100 µg/ml streptomycin (all from Thermo Fisher)] for at least 1 h at 37 °C and 5% CO_2_. After washing with PBS, 3 × 10^5^ cells per well were seeded in triplicates and stimulated either with live pH1N1 or H3N2 viruses (multiplicity of infection of 1), 3 µg/ml ConA (positive control, Sigma-Aldrich), culture medium (negative control), or with one of the pools of peptides at a concentration of 2 µg/well. The pool of peptides for NP1, NP2, M1, NA1, or NA2 are shown in Table 2. After 40-h incubation at 37 °C and 5% CO2, the plates were washed with PBS-T and incubated for 1.5 h at room temperature with 0.25 μg/ml biotinylated anti-pig IFNγ detection Ab (clone P2C11, BD Pharmingen). The cells were washed and incubated with streptavidin-alkaline phosphatase (1:2,000 in PBS, 0.01% Tween 20, 0.1% BSA, Roche, Mannheim, Germany) for 1 h at room temperature. To visualize the spots, 100 μl of 5-bromo-4-chloro-3-indolyl phosphate/nitro blue tetrazolium substrate (Sigma-Aldrich) was added according to instructions of the manufacturer and the reaction was stopped 15–20 min later by using tap water. The spots were counted using the AID ELISPOT reader (AID Autoimmun Diagnostika) and the results from NP1 and NP2 or NA1 and NA2 were pooled and shown as NP and NA, respectively. Results were expressed as IFNγ-producing cell number per 10^6^ stimulated cells after subtraction of the average number of spots in medium stimulated control wells.

**Table 2:** Pools of peptides (15mers overlapping by 10) for each antigen.

Pool	Genbank Accession number	Amino acid residues	Number of peptides
NP1	AKJ82485	1–259	62
NP2	AKJ82485	248–498	62
M1	KR701100.1	1–252	61
NA1	ATE49827	1–239	57
NA2	ATE49827	227–469	58

Intracellular staining (ICS) was performed to analyze IFNγ, TNF and IL-2 production by CD4 and CD8b cells in BAL. Cryopreserved cells from BAL were thawed and seeded in duplicates in 1 × 10^6^ cells per well and stimulated overnight with live pH1H1 or H3N2 (MOI = 1) or medium as a negative control or were stimulated with peptide pools covering NP and M1 proteins included in the vaccine (2 μg/ml) for 5 h at 37 °C and 5% CO_2_, as previously described^34^. One hour following the peptide stimulation, Brefeldin A (GolgiPlug™, BD Biosciences) was added in all wells and 4 h later, the cells were centrifuged for 4 min at 1500 rpm, washed twice with PBS and stained with the primary Abs listed on Table 3. Twenty minutes after the surface staining and incubation at 4 °C, the cells were fixed and permeabilized with BD Cytofix/Cytoperm (BD Biosciences, Cat. No: 555028) as per the instructions of the manufacturer. Cells were then incubated for 30 min at 4 °C with the directly conjugated Abs, afterwards washed twice and finally stained with the secondary rat anti-mouse IgG2a antibody for 20 min at 4 °C. The cytokine production from CD4 and CD8 T cells was analyzed using the flow cytometry antibody panel listed in Table 3. The cells were also stained for Near-Infrared Fixable LIVE/DEAD stain (Invitrogen, Cat. No: L34975), for identification of live cells. The frequency of cytokine production shown is after subtraction of the frequencies found in medium control wells (unstimulated).

**Table 3:** Antibodies used for the intracellular cytokine staining.

Antigen	Clone	Isotype	Fluorochrome	Dilution used	Source of primary Ab	Details of secondary Ab
CD4	74-12-4	IgG2b	PerCP-Cy5.5	1:50	BD Biosciences (Cat. No: 561474)	
CD8b	PPT23	IgG1	FITC	1:200	Bio-Rad Laboratories (Cat. No: MCA5954F)	
TNF	MAb11	IgG1	BV421	1:100	BioLegend (Cat. No: 502932)	
IFNγ	P2G10	IgG1	APC	1:800	BD Biosciences (Cat. No: 561480)	
IL-2	A150D 3F1 2H2	IgG2a	PE-Cy7	1:400 (primary) 1:100 (secondary)	ThermoFisher (Cat. No: ASC0924)	Rat-anti-mouse, IgG2a, BioLegend (Cat. No: 407114)

### Statistical analysis

Statistical analyses were performed using GraphPad Prism 9.2.0 (GraphPad Software, San Diego, CA, United States). The data sets were first analyzed for normality and then subjected to either a one-way or two-way ANOVA and Bonferroni’s multiple comparisons test when normally distributed or to a Kruskal-Wallis test and Dunn’s multiple comparisons test when normality was not achieved (the figure legends state the data sets/ graphs that were not normally distributed). Significant differences were either presented on each graph or listed on Table 1 (*p < 0.05, **p < 0.01, ***p < 0.001, ****p < 0.0001). Until 28 DPI, all animals were treated identically and significant differences between the groups were not identified.

### Reporting summary

Further information on research design is available in the Nature Research Reporting Summary linked to this article.

## Supplementary information

Supplementary Material

REPORTING SUMMARY

## Data Availability

Data generated or analyzed during this study that are critical to the reported findings are available within the article and its Supplementary Information files. Additional supporting data are available from the corresponding authors without undue reservation.
