# Supplementary material for: Immunization with matrix-, nucleoprotein and neuraminidase protects against H3N2 influenza challenge in pH1N1 pre-exposed pigs
Source: NPJ Vaccines. 2023 Feb 15;8:19. doi: 10.1038/s41541-023-00620-2 (PMC9930017; doi:10.1038/s41541-023-00620-2)
Supplement: Supplementary file 1 — Supplementary Material [file 41541_2023_620_MOESM1_ESM.pdf]

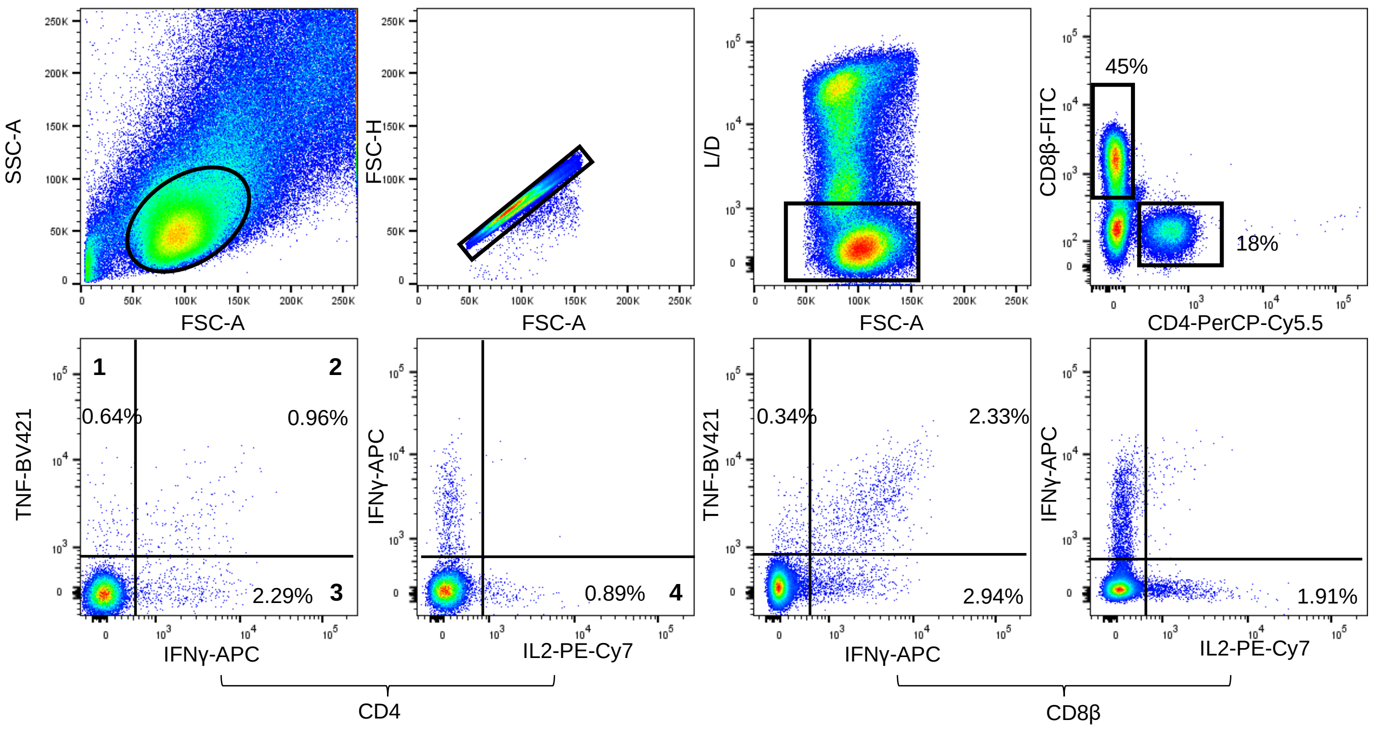

Suppl. Figure 1. Gating strategy followed for analysis of cytokine production by porcine CD4 and CD8 T cells. Cells were stimulated overnight with pH1N1 or H3N2 or for 5 hours with NP2 or M1, followed by intracellular cytokine staining. Lymphocytes were gated by light scatter properties and were further sub-gated for exclusion of doublets and dead cells. Live cells were gated for CD4 and CDβ8 cells and production of TNF (1), IFNγ (3), TNF/IFNγ (2) and IL-2 (4) was determined with the indicated gates. The gates shown are from BAL tissue stimulated with pH1N1.

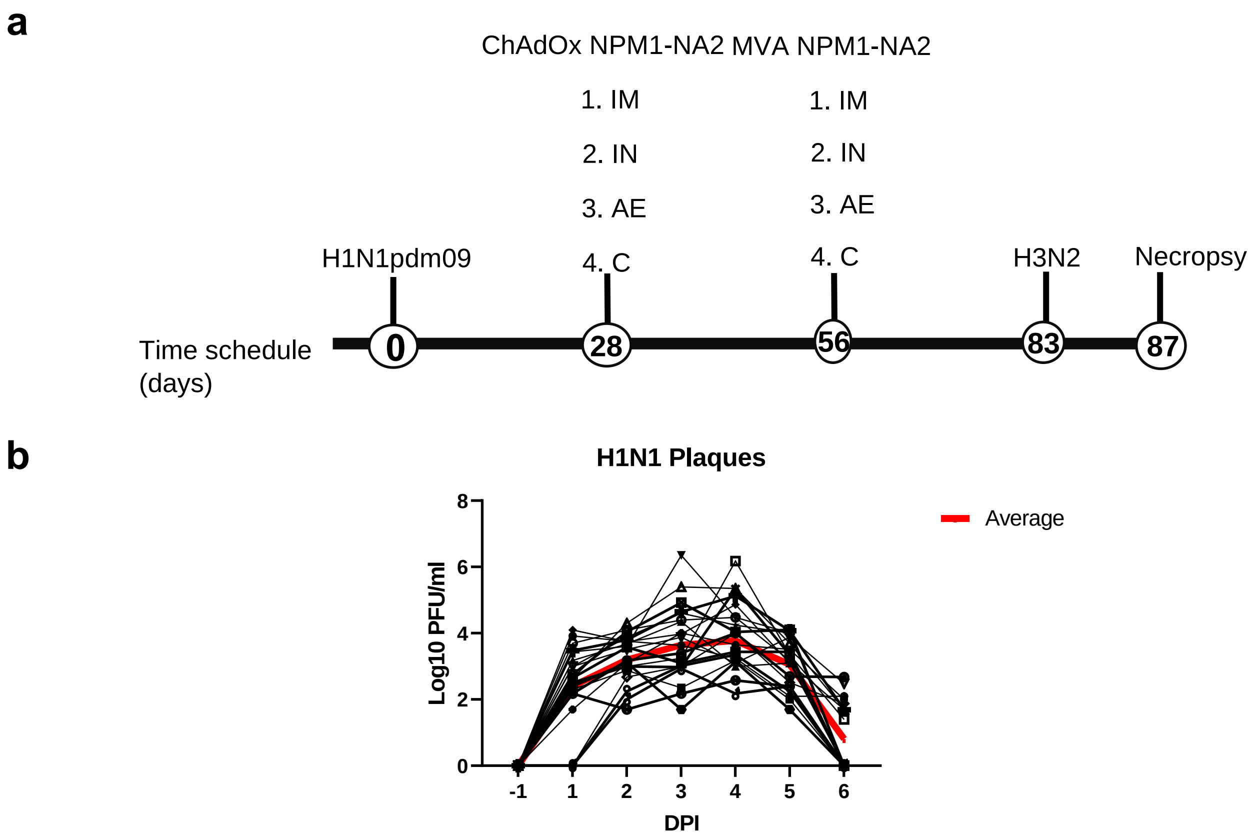

Suppl. Figure 2. Experimental design and pH1N1 viral load for the efficacy study. Twenty-four pigs were infected with pH1N1 Influenza A virus, four weeks later immunized with ChAdOx-NPM1-NA2 intramuscularly (IM), intranasally (IN) or by aerosol (AE) and four weeks later boosted with MVA-NPM1-NA2. After four weeks all pigs were challenged with H3N2 and four days later culled. Control (C) animals were infected but not immunized. Weekly blood samples were collected **(a)**. pH1N1 virus load was determined by plaque assay of daily nasal swabs at the indicated days post infection (DPI) following pH1N1 inoculation on D0 **(b)**.

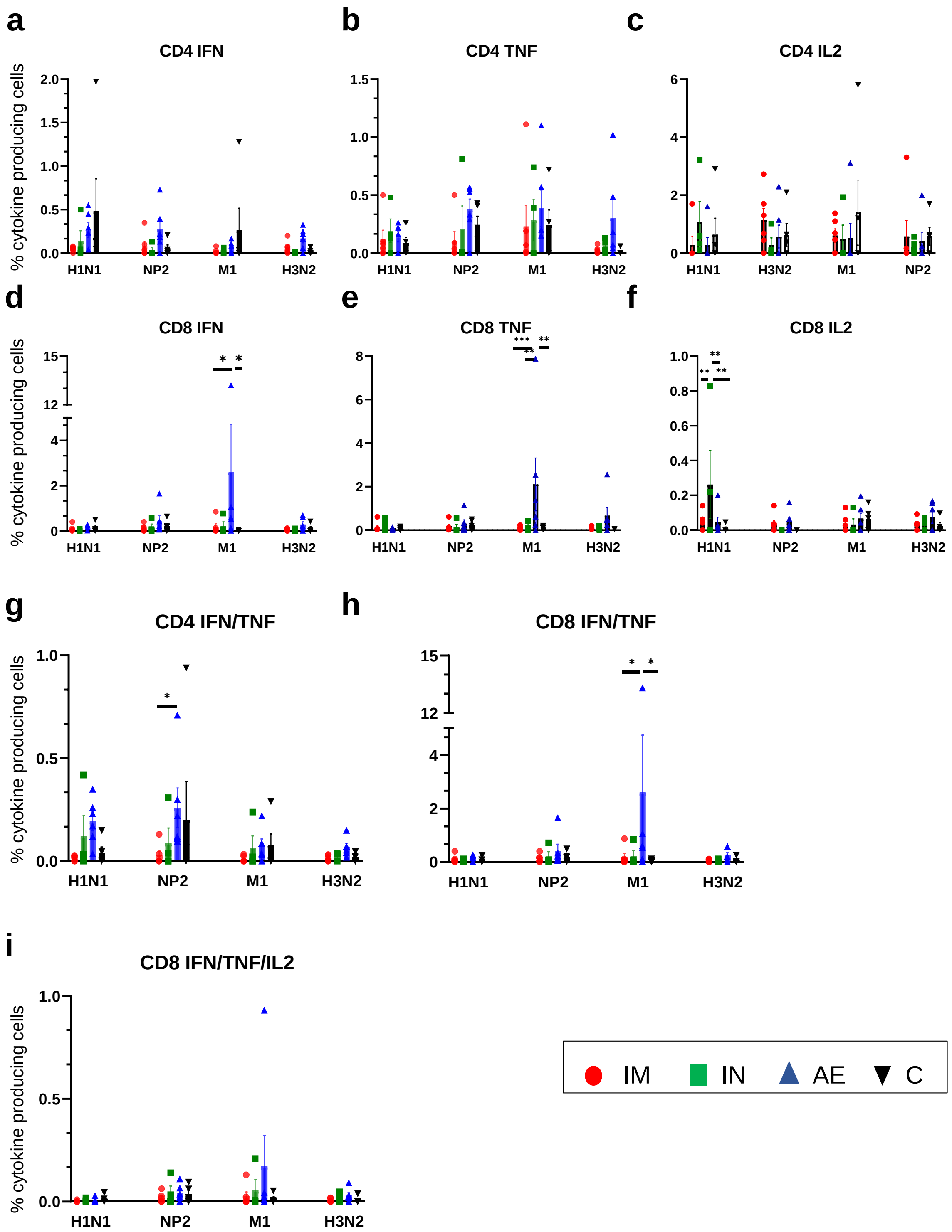

Suppl. Figure 3. T cell cytokine responses in BAL. BAL was collected four days after the H3N2 challenge. Cryopreserved cells from D87 were thawed, stimulated with pH1N1, NP2, M1 or H3N2 and IFNγ, IL-2, TNF, IFNγ/TNF and IFNγ/TNF/IL-2 cytokine secretion was measured in CD4 **(a-c, g)** and CD8 **(d-f, h, i)**. T cells by intracellular cytokine staining. Each symbol represents an individual animal, the top of the bar the mean and the line the standard error (SEM). Two-way ANOVA and Bonferroni's multiple comparisons test were used to compare responses between groups and asterisks indicate significant differences (*p<0.05, **p<0.01, ***p<0.001).
